# Placental syndromes and maternal cardiovascular health

**DOI:** 10.1042/CS20211130

**Published:** 2023-08-22

**Authors:** Helen Casey, Natalie Dennehy, Abigail Fraser, Christoph Lees, Carmel M. McEniery, Kayley Scott, Ian B. Wilkinson, Christian Delles

**Affiliations:** 1 School of Cardiovascular and Metabolic Health, University of Glasgow, Glasgow, Scotland, U.K.; 2 Chelsea and Westminster NHS Foundation Trust, London, England, U.K.; 3 Department of Population Health Sciences, Bristol Medical School, and the MRC Integrative Epidemiology Unit at the University of Bristol, Bristol, U.K.; 4 Division of Experimental Medicine and Immunotherapeutics, Department of Medicine, University of Cambridge, Cambridge, England, U.K.

**Keywords:** cardiovascular health, cardiovascular risk, placenta, preeclampsia

## Abstract

The placental syndromes gestational hypertension, preeclampsia and intrauterine growth restriction are associated with an increased cardiovascular risk to the mother later in life. In this review, we argue that a woman’s pre-conception cardiovascular health drives both the development of placental syndromes and long-term cardiovascular risk but acknowledge that placental syndromes can also contribute to future cardiovascular risk independent of pre-conception health. We describe how preclinical studies in models of preeclampsia inform our understanding of the links with later cardiovascular disease, and how current pre-pregnancy studies may explain relative contributions of both pre-conception factors and the occurrence of placental syndromes to long-term cardiovascular disease.

## Introduction

Cardiovascular disease (CVD) is the leading cause of mortality for women worldwide [1]. However, sex disparities persist with women remaining understudied, underdiagnosed and undertreated [1,2]. Women have been shown to develop CVD on average 10–15 years later than men [2,3]. This presents an opportunity for a period during which at-risk women can be identified and preventative CVD interventions put in place. Sex-specific CVD risk factors allow us a unique opportunity to identify and modify risk in this underserved population. Pregnancy has long been thought of as a stress test for the body [4]. The ‘so called’ placental syndromes, such as gestational hypertension (GH), preeclampsia (PE) and foetal growth restriction (FGR) are recognised risk factors for CVD later in life [5,6].

**Figure 1:**
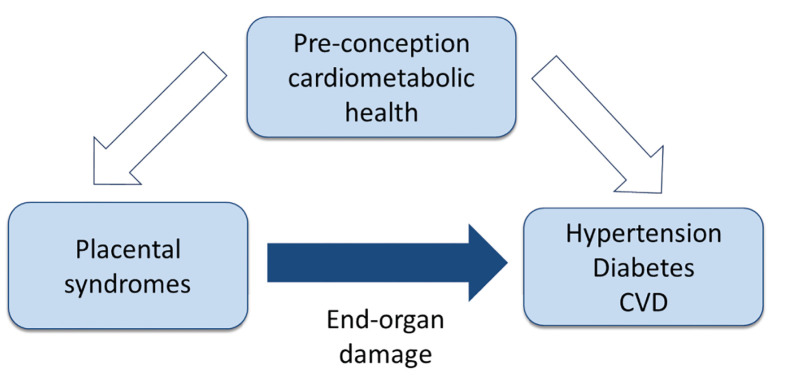
Conceptual design of the relationships between placental syndromes and cardiovascular health

**Figure 2:**
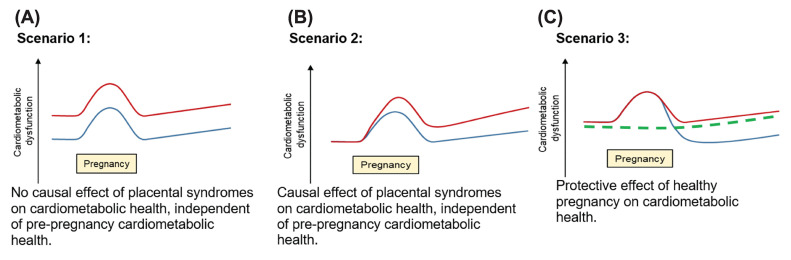
Different hypothetical models of life-course trajectories of cardiovascular dysfunction in healthy versus unhealthy pregnancies and in nulliparous women (**A**) Lifetime risk is predominantly driven by pre-conception cardiovascular health. (**B**) Lifetime risk is predominantly driven by placental syndromes. (**C**) A healthy pregnancy leads to a healthier lifecourse. Red lines: placental syndromes; blue lines: no placental syndromes; dashed green line: nulliparous women.

In this review, we summarise some of the current thinking around the pathogenesis of placental syndromes focusing on PE, one of the most severe forms of hypertensive disorders of pregnancy and also the cause of FGR and preterm birth in many cases, and explore the relationship between pre-conception cardiovascular health, PE and future cardiovascular risk (Figure 1). An important question is whether placental syndromes are the result of suboptimal pre-conception health or whether they develop independently during pregnancy for reasons unrelated to pre-conception health (Figure 2). Whilst in reality both concepts may co-exist and interact, we look at them separately as they have different clinical consequences.

## Placental syndromes and hypertensive disorders of pregnancy

Placental syndromes include GH, PE, FGR, preterm delivery, premature rupture of membranes, placental abruption, pregnancy loss and stillbirth. These syndromes share a common aetiology thought to arise from a complex interaction between defective placentation, trophoblast dysfunction and maternal cardiovascular and multi-system dysfunction [7]. Placental syndromes complicate 10–15% of pregnancies and are a leading cause of maternal and child morbidity [8]. Whilst placental syndromes are considered self-limiting disorders of pregnancy, it is now also well established that they are associated with an increased risk of CVD later in life. A systematic review and meta-analysis by the National Institute for Health and Care Excellence found that PE was associated with a 4-fold increased risk of hypertension, and 2-fold excess risk of ischaemic heart disease and stroke [9]. With regard to GH, a recent systematic review and meta-analysis demonstrated an approximately 1.5-fold greater risk of overall CVD in women who had GH in the first pregnancy, which increased further if more than one pregnancy was affected by GH [10]. A large systematic review has also shown FGR to be associated with a 1.5- to 2.5-fold increased risk of maternal CVD [11]. Collectively, a wide range of pregnancy complications has been found to be associated with increased lifetime risk of CVD [11].

Hypertensive disorders of pregnancy are defined by hypertension existing during pregnancy and up to 12 weeks after delivery [12]. Hypertensive disorders of pregnancy are classified into four types: PE, GH, superimposed PE and chronic hypertension [12,13]. The cardiovascular risks associated with hypertensive disorders of pregnancy are troublingly evident in young populations. Women who develop hypertension in pregnancy have a 12- to 25-fold greater risk of developing permanent hypertension in the year after giving birth, and one-third of women develop hypertension over the subsequent decade [14]. The risks for developing stroke, heart failure, and ischaemic heart disease have been shown to be the highest 1–10 years postpartum [15].

For reasons outlined above, the shared pathogenesis of placental syndromes means that the cardiovascular complications associated with placental syndromes are also similar. In their recent review, Melchiorre et al. [13] have therefore compared cardiovascular outcomes between women with uncomplicated pregnancies and women with hypertensive disorders of pregnancy including the above placental syndromes but also stillbirth and gestational diabetes. They found that hypertensive disorders of pregnancy are associated with increased risk of a wide range of adverse cardiovascular outcomes including hypertension, peripheral artery disease, asymptomatic atherosclerosis, asymptomatic heart failure, heart failure, coronary artery disease and cerebrovascular disease. We share this view of a generally increased cardiovascular risk following complications of pregnancy.

Although most of the hypertensive disorders of pregnancy are captured within placental syndromes, not all women who experience placental syndromes will be hypertensive. Understanding the mechanisms behind the common shared aetiology alongside the differing pathogenesis of the placental syndromes will help us to understand the future maternal cardiovascular risk they convey. In the following sections, we will describe the abnormal placentation in PE that shares features with abnormal placentation in other placental syndromes [16].

## Pathogenesis of preeclampsia

Despite a wealth of research we only begin to understand the complexity of placental syndromes and the multiple mechanisms involved in these conditions. The aetiology of PE is thought of in two stages, firstly abnormal placentation early in the first trimester followed by a second clinical stage in which a release of antiangiogenic and inflammatory factors is associated with maternal widespread endothelial dysfunction, hypertension, and organ damage leading to the clinical syndrome of PE [7,17,18].

This two-stage model translates into a somewhat arbitrary differentiation between early-onset (before 34 weeks) PE (associated with higher risk of intrauterine growth restriction) and late-onset (34 weeks and beyond) PE (frequently associated with maternal obesity and large-for-gestational-age neonates) [19]. Early onset PE is often thought to predominantly represent stage one of the pathogenesis whereas late onset PE is a disease of stage two or the maternal syndrome. However, transcriptional profile studies indicate a common gene signature in the maternal blood for both subtypes [20]. The two subtypes of PE and potentially differing aetiology is another challenge in elucidating the full pathogenesis of PE.

The two-stage model of PE may be seen as oversimplified and in part results from data in experimental models. However, whilst PE is a complex and multifactorial disease some simplification helps us to understand the key pathogenetic principles and thereby develop diagnostic and therapeutic strategies for PE.

### Cardiovascular dysfunction in the preclinical phase of preeclampsia

PE is associated with significant changes in cardiovascular function, which precede the development of the clinical syndrome. In healthy pregnancy, the cardiac output increases and peripheral resistance drops, and these changes begin to occur even at 6 weeks gestation, (several weeks prior to placental development) [21,22]. In PE, there is a disruption of this process, with women who will go on to develop early onset PE (onset <34 weeks of gestation) demonstrating a reduced cardiac output and increased vascular resistance even before conception [23]. This suggests that there is a cardiovascular predisposition to PE prior to pregnancy, rather than these changes reflecting the cardiovascular consequences of impaired placental function.

### Abnormal placentation in the preclinical phase of preeclampsia

During placental implantation, trophoblasts invade deeply into the maternal uterine spiral arteries forming vascular sinuses, high capacitance, high flow vessels [7]. In PE the high resistance maternal circulation and incomplete remodelling of the spiral arteries leads to narrow vessels and subsequent placental ischaemia [24]. These narrow spiral arteries are also prone to atherosis compounding ischaemia [7]. Hypoxia and oxidative stress have a central role in the abnormal shallow placentation of trophoblasts [25–27]. Trophoblast proliferation is favoured by low oxygen tension environment, prior to invasion trophoblasts plug the tips of the spiral arteries [25]. These plugs collapse forming the vascular sinuses. The increasing oxygen tension in maternal blood causes oxidative stress and promotes differentiation to the invasive trophoblast phenotype which remodel the spiral arteries [27,28]. Intermittent hypoxia and reoxygenation caused by poor spiral artery invasion however may result in excess oxidative stress [28]. Placentas from women with PE show an imbalance of reactive oxygen species (ROS)-generating enzymes and antioxidants [7,18]. *Ex vivo* preeclamptic trophoblast ROS-producing enzyme expression and activity are increased and inhibit the signalling pathway that promote trophoblast invasiveness [26]. Oxidative stress also promotes the transcription of antiangiogenic factors such as soluble fms-like tyrosine kinase 1 (sFlt-1) [29]. In placentas from those with PE antioxidant mechanisms are impaired with decreased expression of superoxide dismutase and glutathione peroxidase compared with women with normal pregnancies [30]. Hypoxia-inducible factor-1α (HIF-1α), a marker of cellular oxygen deprivation, is expressed at high levels in proliferative trophoblasts in the placentas of women with PE [26]. Overexpression of HIF-1α in pregnant mice is associated with hypertension, proteinuria, FGR and failure of invasive trophoblastic [31,32]. Inhibition of HIF-1α by 2-methoxyestradiol, a metabolite of estradiol, suppresses the production of sFlt-1, an antiangiogenic factor known to play a central role in stage II of PE [33].

Uterine natural killer (uNK) cells have also been suggested to play a role in the abnormal placentation [7,18]. uNK cells regulate trophoblastic invasion and the depth of spiral artery remodelling [34]. uNK cells recognize maternal and paternal major histocompatibility complexes (MHCs) [35]. They express KIR (killer cell Ig-like receptors), while trophoblasts express the main KIR ligand, polymorphic HLA-C (human leukocyte antigen-C) MHCs [36]. Pregnancy results in a unique combination of KIR and HLA-C which may affect the success of placentation [35,36]. Mouse models have shown that inhibition of the uNK response by MHC-self recognition leads to defective artery remodelling [37]. Certain uNK KIR haplotypes appear protective against PE while others confer risk [35,38,39].

Abnormalities of placentation have been found not only in the spiral arteries; atherosclerotic changes in the radial arteries that supply the decidua have also been observed [40]. The presence of decidual vasculopathy (DV) lesions in PE are associated with worse clinical outcome, higher diastolic blood pressure, worse renal function and foetal death [41,42]. These preeclamptic DV decidua show signs of oedematous endothelium, hypertrophy of the vessel media, and loss of smooth muscle modifications [40,42]. It is currently unclear whether decidua changes in PE represent widespread endothelial damage or are part of the first stage of PE pathogenesis during abnormal placentation [7]. However, stromal cells from women with a history of severe PE fail to decidualize *in vitro* and their conditioned medium failed to support cytotrophoblast invasion [43]. Global transcriptional profiling of decidual tissue from women with PE has also shown defects in gene expression [43]. This suggests that abnormal placentation of stage one of PE pathogenesis might be the result from defects in both the invasive trophoblast and decidua. Some have proposed that PE – and particularly early onset PE or PE accompanied by FGR – is caused by impaired placentation and spiral artery remodelling. More recently, Melchiorre et al. [44] have suggested that PE is a consequence of pre-existing adverse maternal cardiac function and haemodynamics that fail to adapt to the increasing cardiometabolic demands of pregnancy, resulting in placental hypoperfusion and PE, and it is plausible that the abnormal trophoblastic invasion is predisposed by pre-existing cardiovascular dysfunction [23].

### Clinical syndrome of preeclampsia

The second stage of PE associated with the clinical syndrome is linked to an increased release of anti-angiogenic factors such as sFlt-1. sFlt-1 is a soluble protein that exerts antiangiogenic effects by binding to and inhibiting the biological activity of proangiogenic proteins vascular endothelial growth factor (VEGF) and placental growth factor (PlGF). VEGF is important for the maintenance of endothelial cell function in which dysfunction of in the brain, liver, and glomeruli leads to the clinical syndrome of PE [45]. PlGF, is a member of the VEGF family, is important in angiogenesis and selectively binds to VEGF Receptor 1/sFlt-1 [46]. In PE, sFlt-1 protein levels are high in maternal plasma, and sFLT1 mRNA expression is also high in PE placentas [45,47]. Injecting exogenous sFlt-1 protein into rodents induces a PE like state with hypertension, proteinuria and glomerular endotheliosis [48]. Depletion of sFlt-1 in preeclamptic plasma using antibodies in animal models reduces the clinical symptoms and signs of PE and resolution of signs and symptoms when sFlt-1 levels are lowered by 50% [49–51]. Soluble endoglin (sENG), another anti angiogenic factor acting as a transforming growth factor β1 inhibitor is elevated in the serum of women with PE 2 months before the onset of clinical signs of PE and correlates with disease severity [52,53]. In rodent models, it appears to increase the effects of sFlt-1, inducing a severe PE like state in pregnancy [52,54].

The maternal syndrome of PE is a proinflammatory state. Many immune changes driving this have been established in PE. A reduction in interleukin 10 (IL-10) and increase in proinflammatory cytokines has been found in PE. IL-10 is a cytokine that causes the differentiation of the T cell into the Th (T helper type) 2 phenotype [55–57]. IL-10 neutralises proinflammatory cytokines, AT1-AA (angiotensin II receptor 1 autoantibodies) and placental reactive oxygen species [55]. Th2 polarization occurs in pregnancy and is characterized by a shift in T-cell phenotype towards Th2 relative to Th1 [58,59]. An aberrant shift towards the Th1 phenotype in PE has been found [59]. A PE like syndrome can also be induced in normal pregnant rats with transfer of CD4+ cells obtained from reduced uterine perfusion pressure (RUPP) models [60]. PE is associated with elevated complement levels [61]. In animal models, complement inhibition restores spiral artery capacitance and decreases sFlt-1 production [62,63]. In severe PE where haemolysis elevated liver enzymes low platelets (HELLP) syndrome has occurred, the complement pathway is dysregulated [64]. HELLP syndrome shares a genetic mutation with atypical haemolytic uraemic syndrome, caused by uncontrolled complement activation [64]. The presence of AT1-AA in the sera of preeclamptic women has been established in PE [65]. AT1-AA can stimulate placental production of antiangiogenic factors such as sFlt-1 [66]. CD19+CD5+ cells alongside AT1-AA, are elevated in sera of women with PE [67]. This suggests AT1-AA may be made by a subpopulation of CD19+CD5+ and a role of B cells in PE's maternal syndrome [67].

Established PE is associated with profound cardiovascular system changes in comparison to healthy pregnancies, the nature of which varies according to the timing of onset of PE and the presence of FGR. Early onset PE is associated with a lower cardiac output and increased peripheral resistance, whereas PE developing after 34 weeks is associated with a higher cardiac output and lower peripheral resistance [68]. These changes are present both before and after the development of the clinical syndrome, with high resistance and low cardiac output also seen in women with FGR in the absence of hypertension [69]. Later studies demonstrated that irrespective of the timing of onset of PE, the presence of FGR was associated with low cardiac output and increased peripheral resistance, and the absence of FGR with high cardiac output and low peripheral resistance [70,71]. Preterm PE is also associated with a greater degree of cardiac dysfunction, in particular diastolic dysfunction, and this is seen to persist in the postnatal period and is even detectable at one year postpartum [68,72].

The causes of PE are complex and have long been the subject of debate. The observation that histological changes could be seen in the placenta of patients with PE led to the focus of research on the placenta as the source of the problem, although, the presence of these lesions is by no means universal [73]. Certainly, PE can be associated with other syndromes associated with impaired placental function such as FGR and can share pathophysiological features [74]. However, the hypothesis that the placenta is the primary organ responsible for PE belies the evidence that many of the cardiovascular changes of PE not only persist in the postpartum period after the placenta is delivered, but in fact precede the development of the placenta by a number of weeks. Thus, PE can be seen as a cardiovascular disorder, with abnormal placentation being associated, though not necessarily causative, of the clinical syndrome [44].

A specific example is the presence of acute atherosis as in placentas of women with placental syndromes. These arterial lesions are specific to the spiral arteries at the fetal–maternal border and are thought to be multifactorial in nature but mainly driven by inflammatory processes. It is not unreasonable to propose that women who are prone to such vascular lesions in the placenta are at higher risk of vascular complications later in life that are also often driven by inflammatory mechanisms. This concept has recently been reviewed by Pitz Jacobsen et al. [75].

## Treatment

The treatment of PE primarily focuses on three areas – prevention of the clinical syndrome of hypertension and proteinuria, the treatment of hypertension and the prevention of seizures.

The use of low dose aspirin in the early second trimester of pregnancy results in a significant reduction in the incidence of PE in women at increased risk [76]. Aspirin has a number of effects, including inhibition of thromboxane and lipid peroxidases, and represses sFlt-1 production [77,78]. However, aspirin has been demonstrated to improve cardiovascular function in a wide range of cardiovascular conditions including stroke and myocardial infarction [79], and thus the effect of aspirin may be more general. Other therapies have been trialled for prevention of PE including calcium supplementation with limited evidence suggesting a possible benefit [80].

Treatments for hypertension in PE have been largely unchanged over the last 50 years and are often used ‘off licence’ due to their long history of use, despite enormous changes in strategies to treat hypertension in the non-pregnant population. Adrenoceptor antagonists, such as labetalol and doxazosin, calcium channel blockers and dopamine antagonists such as alpha-methyldopa form the mainstay of antihypertensive therapy during pregnancy.

Magnesium sulphate infusion or bolus is near-universal first line therapy for the prevention and treatment of seizures (eclampsia). Its mechanism of action is varied, is thought to act as a central vasodilator, causing cerebral vasodilation and preventing cerebral ischaemia. It is also thought to have a cerebro-protective effect for the fetus if given shortly before delivery.

The definitive treatment for PE is delivery and hence iatrogenic preterm birth is common, usually indicated because of uncontrolled hypertension, organ dysfunction or risk of seizures. Treatments that act as disease-modifying treatments, rather than merely symptom control, would prolong gestation by safely allowing expectant management of PE. Sildenafil prolonged gestation by a non-significant 4 days gestation [81]. This difference may have been due to sildenafil’s effect as an antihypertensive as more women in the control group were delivered for uncontrolled hypertension. The use of sildenafil in clinical practice has subsequently been curtailed due to the findings of increased neonatal death due to pulmonary hypertension seen in trials for its use in FGR [82,83]. No benefit in terms of prolongation of gestation has been seen from the use of esomeprazole [84], pravastatin [85,86] or antithrombin [87]. There is evidence to support the prolongation of gestation in expectantly managed PE with metformin, although more studies are needed [88]. Nitric oxide donor S-nitrosoglutathione has demonstrated efficacy in improving proteinuria, augmentation index and platelet function in women with severe early onset PE, suggesting a disease-modifying mechanism of action, although further studies are required to investigate any pregnancy prolongation [89–91].

### Animal models of preeclampsia

Though our understanding of the pathological mechanisms of PE are limited, preclinical models have proven to be an invaluable tool in investigating PE. There are a wide variety of rodent models designed to mimic specific symptoms or underlying factors of PE. One such model uses arterial clips to physically inhibit uterine blood flow. In the RUPP model, pregnant rodents undergo lower abdominal and ovarian artery occlusion at mid-gestation [92]. This leads to the *de novo* development of hypertension, proteinuria, FGR and impaired renal and vascular function [92–94]. RUPP rodents also exhibit an increased inflammatory state, which has been indicated in PE development [55–59].

Genetic models of PE have also become popular among researchers. Genetic knock-out models targeting the inflammatory aspect of PE have been developed including deficiencies in IL-10, IL-4 and complement components [93,95]. Other models result in the overexpression of target genes such as STOX1 (storkhead box 1), a transcription factor expressed in extravillous trophoblasts, supported by evidence of a high-risk allele variant [96,97]. The stroke-prone spontaneously hypertensive rat (SHRSP) and BPH/5 (borderline hypertensive 5) mouse strains have been utilized as genetic linkage models of PE with both developing hypertension in adult life that worsens during pregnancy alongside the other clinical symptoms of PE observed in humans [98,99].

Hypertensive disorders of pregnancy can be mimicked pharmacologically by the administration of substances known to cause vascular dysfunction. Pregnant Sprague-Dawley rats injected with adenoviral sFlt-1 exhibited significant maternal symptoms of PE in a dose-dependent manner related to disease severity, as observed with varied severities in humans [100]. However, the usefulness of this model has been debated with some studies showing evidence of abnormal vascular function post-partum as early as 2 months and others at 8 months post-partum [101,102]. A more relevant model is a transgenic inducible human sFlt-1/reverse tetracycline-controlled transactivator (hsFLT1/rtTA) mouse, which overexpresses sFlt-1 ubiquitously throughout pregnancy [103]. Other pharmacological intervention models of PE seek to induce a PE phenotype rather than target a specific biomarker by administering pro-inflammatory and/or vasoconstrictive agents. One of these is the L-NAME (Ω-nitro-_L_-arginine methyl ester) model, which replicates endothelial dysfunction by inhibiting nitric oxide synthase (though there is no confirmation that nitric oxide plays a role in PE pathogenesis) and is commonly used to study therapeutic agents [104].

Due to the known involvement of the renin–angiotensin–aldosterone system (RAAS) in pregnancy and in PE, alterations to this system have been utilised as a means to model PE in rodents. The chronic AT_1_-AA excess model was created by injection of purified rat AT_1_-AA into Sprague-Dawley rats in late gestation which resulted in an observed hypertension and elevated sFlt-1 and sEng (soluble transforming growth factor β [TGFβ] co-receptor endoglin) [105]. However, there was no effect on fetal and placental outcomes in this model. The generation of AT_1_-AA during PE highlights the importance of angiotensin II (ANGII). Patients suffering from PE have been found to have an increased sensitivity to ANGII which may contribute to maternal vascular, placental and renal dysfunction [106]. When delivered to normotensive rodents, ANGII induces a PE-like phenotype that includes classical increased blood pressure alongside placental inflammation, FGR and increased cardiovascular stress [107,108]. By delivering ANGII during pregnancy in the SHRSP rat, dams experience a worsening of pre-existing hypertension and proteinuria as well as impaired uteroplacental flow, renal pathology and changes in placental gene expression as seen in human superimposed PE [107]. The pregnant female human angiotensinogen (hAGN) transgenic rat mated with the male human renin (hREN) transgenic rat is a complex transgenic model which allows detailed dissection of the role of the RAAS in PE [109].

The use of rodent models in the study of PE has many advantages. Both humans, rats and mice share a haemochorial placenta with only a few differences between strains in gross structure and organisation, with the rat being more similar to humans than the mouse [110]. Additionally, both rats and mice have a relatively short gestation period, are easily housed and are more economically viable than non-human primates [111]. However, these models have their drawbacks. The RUPP model surgery is precise with respect to the location of the arterial clips, even a slight shift in placement of the clips can lead to a failure to develop hypertension. Additionally, the surgical method of RUPP does not capture the involvement of trophoblast invasion, immune mechanisms or vascular dysfunction [94]. Genetic models whilst useful cannot capture the polygenic, multi-factorial nature of PE and some express concerns of evolutionary divergence between humans and rodents [112].

## Placental syndromes and maternal cardiovascular risk

The maternal increased risk of CVD associated with a history of placental syndrome is no longer disputed and recognised in clinical guidelines [113]; however whether the placental syndrome is a cause or manifestation of poor cardiovascular health pre-pregnancy leading to a cardiovascular maladaptation to pregnancy remains to be proven (Figure 1).

Many traditional cardiovascular risk factors (adiposity, diabetes, chronic kidney disease, etc.) are shared with the pre-pregnancy risk factors associated with an increased risk of placental syndromes [42]. Most risk factors for PE (including maternal age, nulliparity, body mass index [BMI] and multiple pregnancy) are linked to a higher blood pressure at 8 weeks of pregnancy and a higher rate of rise in blood pressure in the second half of pregnancy compared with women who *do not* develop hypertension in pregnancy, suggesting a continuum of risk in all pregnancies [114]. This gives weight to the school of thought that pregnancy is a stress test that unmasks pre-existing poor cardiovascular health (Figure 2A), rather than a complication of pregnancy causing maternal CVD later in life (Figure 2B), though these two postulated mechanisms are not necessarily entirely mutually exclusive. Pre-conception studies are necessary to answer this question.

Reports using data from the Norwegian Trøndelag Health (HUNT) Study showed that the association between PE and postpartum blood pressure and BMI was attenuated by adjustment for corresponding pre-pregnancy values (by ∼50% and ∼90%, respectively) [115] and that women with PE or GH had higher blood pressure and adverse lipids prior to pregnancy and that this effect persisted until at least the age of 50 [116]. These findings extend to other placental syndromes such as preterm birth and small for gestational age [117]. However, in HUNT there was a long and variable interval between pre-pregnancy measurements and pregnancy (up to 20 years) and a limited panel of cardiometabolic risk factors available. In addition to a potential detrimental effect of placental syndromes on maternal postpartum cardiovascular health, some evidence suggests that a healthy pregnancy may have a lasting protective effect when compared to nulliparity [118,119] (Figure 2C). A large pre-conception study is necessary to help us understand the relationship between pre-conception cardiovascular health, pregnancy and placental syndromes.

Early pregnancy loss may be conceptualised as a severe form of pregnancy complication and/or maladaptation [120]. The pooled risk of miscarriage across pregnancies is 15.3% [121], and many miscarriages missed because they occur very early post-conception. Previous data indicate that women who suffer early spontaneous pregnancy loss have an increased risk of CVD in later life and an increased risk of placental syndromes in subsequent pregnancies [122,123]. However, it is not known whether *pre-pregnancy* cardiovascular health is associated with early pregnancy loss.

Haemodynamic abnormalities in women with established placental syndromes have been reported – typically blood pressure and peripheral resistance are elevated compared with uncomplicated pregnancies [33,68,69,71]. Whether these abnormalities are related to pre-pregnancy cardiovascular risk profiles is unknown, but evidence suggest that abnormal maternal cardiovascular adaptation occurs early in pregnancies that are later complicated by placental syndromes. Mean arterial pressure in early pregnancy is a good predictor of subsequent PE and GH, and first-trimester diastolic pressure is associated with the risk of subsequent FGR, even in the ‘normotensive’ range [124–126]. Previous data also indicate that first trimester haemodynamics may differentiate subsequent PE from GH [127]. Abnormal cardiac function, particularly diastolic dysfunction, may also precede the development of placental syndromes [128]. Although most of these studies are based on small sample sizes and/or contain only small numbers of women who experienced a placental syndrome, they suggest that cardiovascular maladaptation to pregnancy may represent a biomarker of women at risk of developing placental syndromes. This is regardless of whether the maladaptation is driven by pre-pregnancy cardiovascular dysfunction or risk factors, defective placentation, or a combination of the two.

Four small studies have assessed cardiovascular haemodynamics from pre-pregnancy, but the largest included only 45 women [129–132]. One of these reported elevated pre-pregnancy vascular stiffness in women who developed PE [130], and another that healthy pregnancy was associated with a reduction of vascular stiffness between pre-pregnancy and 30 months later [131]. We have conducted two small studies from pre-pregnancy, which, although larger, only yielded 10 cases of PE, which is consistent with the incidence of PE in a healthy population [23,133]. We reported a positive association between the increase in cardiac output from pre-pregnancy to the second trimester and birth weight in healthy women, consistent with previous observations of a high resistance, low output state in FGR pregnancies [134]. We have also shown that women who develop PE and/or FGR have a lower cardiac output and higher systemic vascular resistance pre-pregnancy, although this observation requires confirmation in a larger study [23].

## Direct effect of placental syndromes on long-term cardiovascular disease risk

Whilst the above data provide a link between cardiovascular health, maladaptation during pregnancy and future cardiovascular risk it should be noted that irrespective of pre-pregnancy confounding, many of the mechanisms identified in the pathogenesis of placental syndromes such as PE could potentially provide a direct link to long term cardiovascular risk in women. Exposure to anti angiogenic factors which mediate endothelial dysfunction such as sFlt-1, found to be increased in PE, could lead to longer-term endothelial dysfunction or vascular rarefaction.

Such experience has been made in patients treated with anti-VEGF drugs, a growing class of chemotherapy drugs used in the treatment of solid cancers and age-related macular degeneration. Hypertension occurs in up to 80% of patients on systemic anti-VEGF therapy and nearly all patients taking these drugs experience a rise in blood pressure, even if not to hypertensive levels [135]. Substantial evidence exists to show that chronic VEGF inhibition causes capillary rarefaction, both in preclinical models and in humans [135]. sFLT-1 binds VEGF in a mechanism analogous to anti-VEGF drugs, resulting in inhibition of VEGF signalling. It is plausible that the exposure to antiangiogenic principles during preeclamptic pregnancy can lead to similar long-term vascular changes as observed in therapeutic use of anti-VEGF drugs.

## Perspectives

Allowing understand of how pre-conception cardiovascular risk interacts with placental syndromes may help us to elucidate placental syndromes aetiology. The pathogenesis of placental syndromes, including PE, stem from defective placentation and maternal multi system dysfunction. Placental syndromes may be a consequence of adverse, yet sub clinical pre-conception cardiovascular health. However, if placental syndromes are not linked to pre-conception health, then it would suggest the placental syndromes themselves play a casual role in the development of future cardiovascular risk.

We appreciate that both concepts, i.e. a concept of pre-conception subclinical CVD and a trigger during pregnancy, will play a role in the development of placental syndromes and their long-term consequences for affected women. We presented clinical data on an association between traditional cardiovascular risk factors and the risk of developing PE and other placental syndromes but we also highlighted that removal of the placenta cures or at least dramatically improves the clinical symptoms of PE. How exactly pre-conception risk and any further factors during pregnancy interact remains, however, unknown.

We are currently undertaking an observational, prospective study of healthy, nulliparous women, recruited pre-pregnancy to test the hypothesis that placental syndromes adversely affect cardiovascular health postpartum, independently of women’s pre-conception cardiovascular health. We will assess cardiovascular risk factors and validated intermediate phenotypes for CVD both pre- and post-pregnancy and determine the effect of placental syndromes on post-partum maternal cardiovascular health, independently of pre-pregnancy cardiovascular health. We will also assess the early maternal haemodynamic adaptation to pregnancy to determine whether this is a biomarker for the later development of placental syndromes and the extent to which pre-pregnancy cardiovascular health is linked to cardiovascular maladaptation. Finally, we will also include women who do not intend to conceive during their involvement in the study, allowing us to test, definitively, whether pregnancy *per se* has any effect on future cardiovascular health.

Regardless of the aetiology and answer to this question, women with a history of placental syndromes have been clearly identified as being at high risk for future CVD. Currently we lack the understanding of the aetiology of these women's placental syndromes and subsequent CVD risk but we do not lack the knowledge to put in place preventative CVD initiatives to help reduce risk. Consensus guidance and implementation on long term follow up, along with education for health care professionals and the public on the increased risk associated with a history of placental syndromes is lacking.

If a link between preconception sub clinical CVD and placental syndromes is established, then there is an important opportunity to intervene which does not present itself in pregnancy. Strategies to improve maternal and fetal health must also include public health strategies and targeting those planning to conceive with respect to optimisation of cardiovascular health preconception. Improving the cardiovascular health of women pre-pregnancy, could lead to a reduction in the risk of placental syndromes and the burden of associated long term health risks.

## Data Availability

NA

## Abbreviations

BMI: body mass index
CVD: cardiovascular disease
FGR: fetal growth restriction
hAGN: human angiotensinogen
RAAS: renin–angiotensin–aldosterone system
ROS: reactive oxygen species
sENG: soluble endoglin
